# Prominent hyperintense areas in swollen optic pathway: An indicator of congestive glymphatic pathway?

**DOI:** 10.1016/j.radcr.2022.05.052

**Published:** 2022-06-11

**Authors:** Kiyotaka Kuroda, Satoshi Tsutsumi, Hiroki Sugiyama, Natsuki Sugiyama, Hideaki Ueno, Hisato Ishii

**Affiliations:** Department of Neurological Surgery, Juntendo University Urayasu Hospital, 2-1-1 Tomioka, Urayasu, Chiba, 279-0021, Japan

**Keywords:** Optic pathway, Hyperintense areas, Glymphatic pathway, Congestion

## Abstract

A 34-year-old woman presented with progressive visual impairment for 2 months. Computed tomography and magnetic resonance imaging (MRI) revealed a suprasellar cyst. The patient underwent endoscopic endonasal surgery, which resulted in gross total resection with a diagnosis of craniopharyngioma. Postoperatively, the patient's visual function improved. The constructive interference steady-state (CISS) sequence revealed a remarkable resolution of hyperintense areas in the swollen optic nerve and tract. A 36-year-old woman sustained an abrupt visual loss. She had been diagnosed with cavernous malformations (CMs) in the right frontal lobe and suprasellar region 11 years ago, with an intact optic tract. Cerebral MRI at presentation revealed marked enlargement of the suprasellar CM and swelling of the right optic tract, accompanied by predominant hyperintense areas in the optic pathway. The patient's visual function showed significant improvement with conservative management. The CISS sequence performed 3 months later showed remarkable resolution of hyperintensity in the optic pathway. Prominent hyperintense areas in the optic pathway may indicate congestion of glymphatic flow. Reduction in such areas may be a radiological biomarker reflecting the improvement of visual impairment.

## Introduction

The complex and dynamic fluid-filled channels formed in the perivascular and interstitial spaces of the central nervous system are currently believed to be the glymphatic pathways that channel extracellular fluid and clean metabolites and peptides for optimizing neurological function [1]. Evidence suggests that the optic nerve may function as a glymphatic pathway [2], [3], [4], [5], [6]. Recently, studies using magnetic resonance imaging (MRI) have delineated the possible glymphatic pathways in the intracranial and intraorbital segments of the human optic nerve [7,8].

Craniopharyngiomas are rare benign epithelial tumors that arise along the path of the craniopharyngeal duct and occur in the sellar and suprasellar regions. They commonly cause neurological, endocrinological, and visual symptoms [9]. Tumor-associated edema of the optic tract has been documented as a pathognomonic finding of craniopharyngiomas that are likely to have irregular boundaries with the nerve [10], [11], [12].

Cavernous malformations (CMs) are hamartomatous vascular malformations with high affinity to the veins. CMs of the optic pathway are rare. In general, it is thought that gross total resection of symptomatic CMs can lead to favorable outcome [13].

Here, we report 2 patients presenting with predominant hyperintensities in the optic pathway that appeared in association with a craniopharyngioma and CM and showed remarkable regression with improvement of visual impairment.

## Case report

**Fig. 1:**
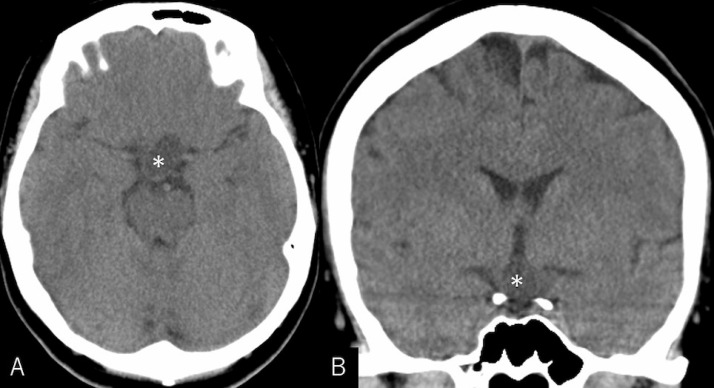
Noncontrast axial (A) and coronal (B) computed tomography scans showing a cystic mass in the suprasellar region (asterisk). It appears as a homogenous hypodense mass lacking intralesional calcifications.

**Fig. 2:**
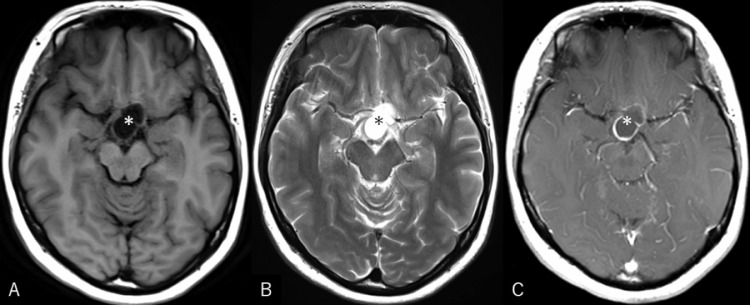
Axial T1- (A), T2- (B), and postcontrast T1-weighted (C) magnetic resonance images demonstrating the cyst, 25 mm × 15 mm in maximal dimension, which exhibits low intensity on T1- and high intensity on T2-weighted imaging with marginal enhancement (A-C, asterisk).

**Fig. 3:**
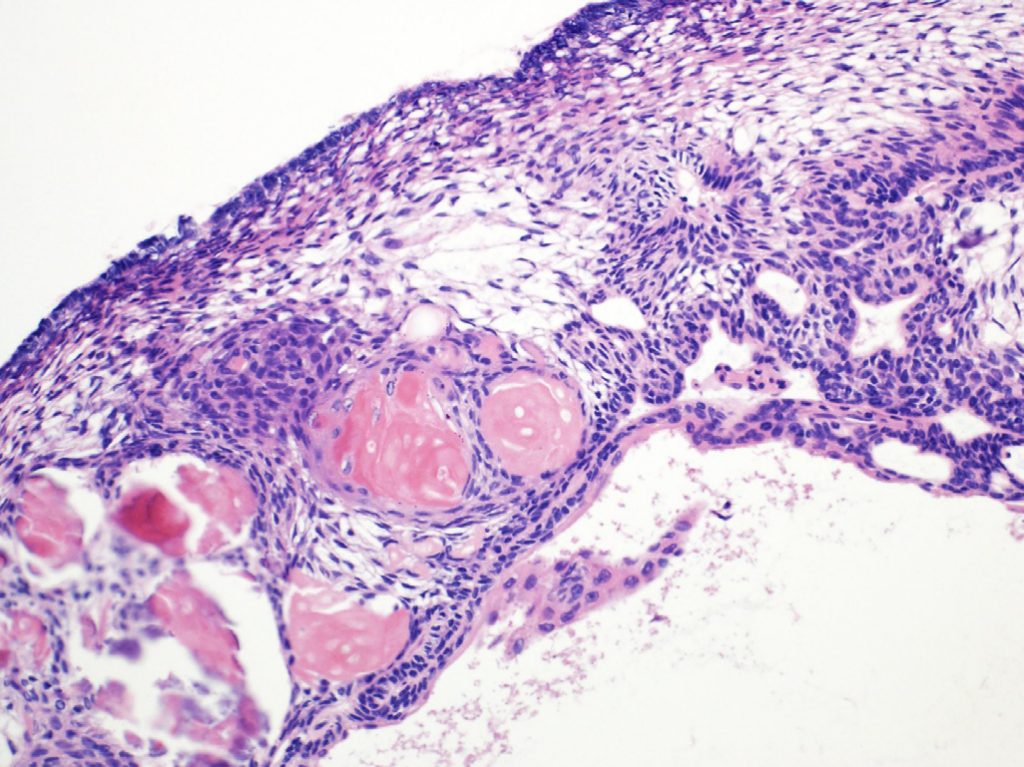
Photomicrograph of resected cyst wall showing proliferation of squamous epithelial cells and depositions of keratin, consistent with adamantinomatous craniopharyngioma. Hematoxylin and eosin stain, ×100.

**Fig. 4:**
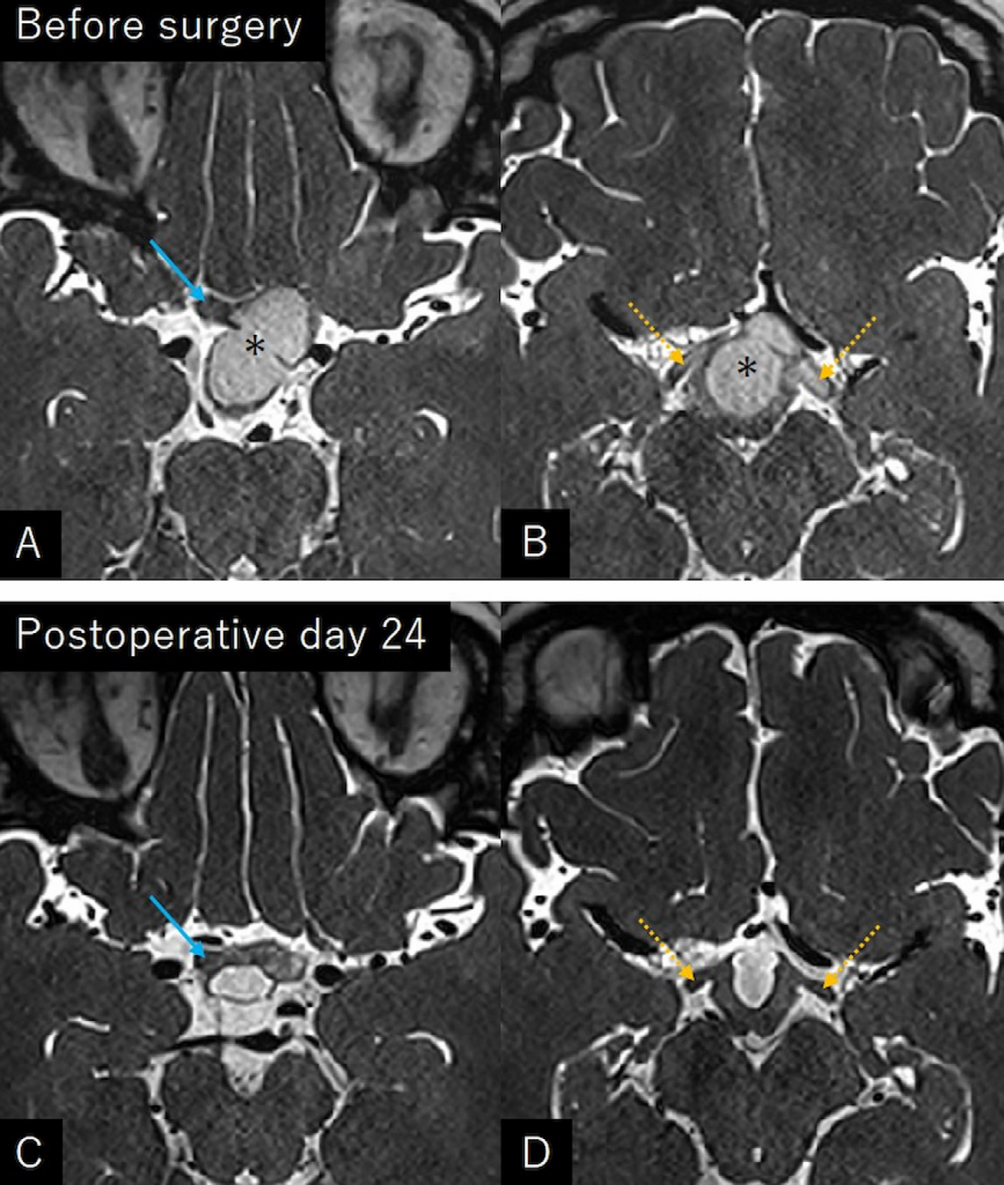
Constructive interference steady-state imaging at the level of the anterior limit of the optic chiasm (A, C) and the optic tracts (B, D) showing remarkable postoperative resolution of the hyperintense areas in the right optic nerve (arrow) and bilateral optic tracts (dashed arrows). Asterisk: tumor.

**Fig. 5:**
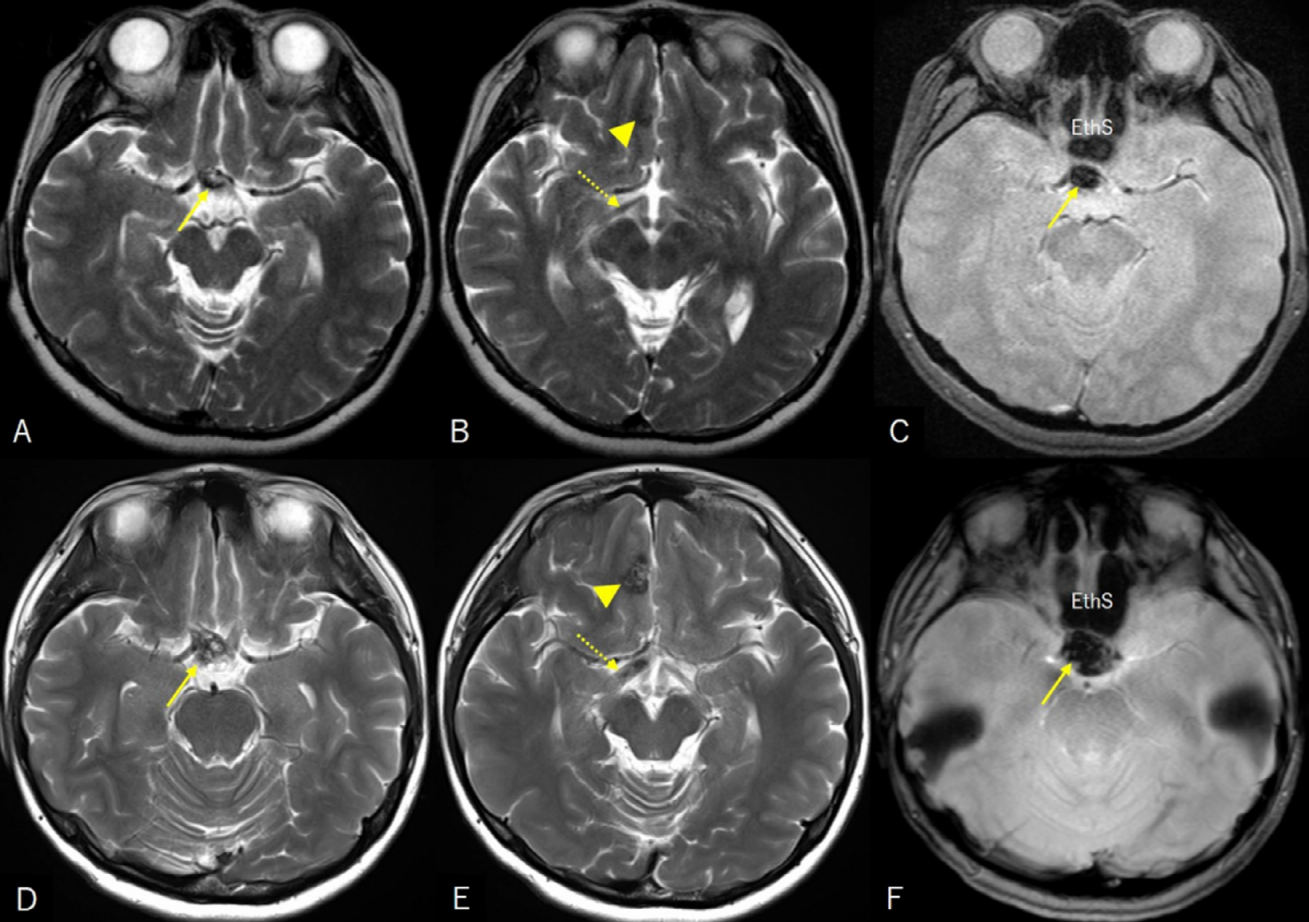
(A-C) Axial T2- (A, B) and gradient-echo T2*-weighted (C) magnetic resonance images performed 10 months before presentation showing a low intense mass in the suprasellar region (A, C, arrow), in addition to a cavernous malformation in the right frontal lobe (B, arrowhead). The right optic tract appears intact (B, dashed arrow). (D-F) Axial T2- (D, E) and gradient-echo T2*-weighted (F) magnetic resonance images at the presentation showing a marked enlargement of the suprasellar mass and swelling of the right optic tract. Cavernous malformation in the right frontal lobe does not show any signs of recent hemorrhage (E, arrowhead). A, C, D, and E are images near the same level. EthS, ethmoid sinus.

**Fig. 6:**
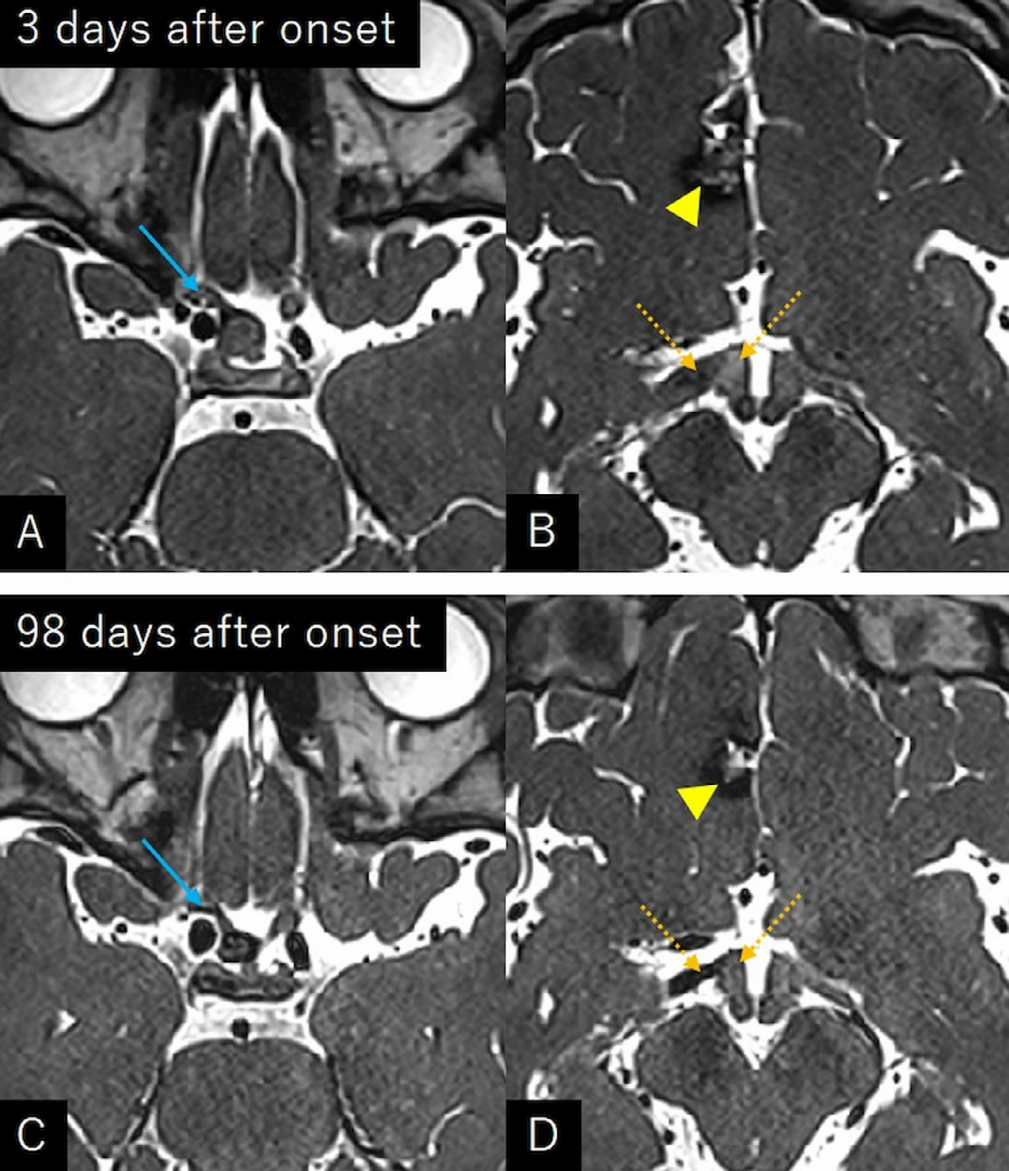
Constructive interference steady-state imaging at 3 days after onset (A, B) and at 98 days after onset (C, D) showing resolution of hyperintense areas in the right optic nerve (A, C, arrow) and the optic tract (B, D, dashed arrows). Arrowhead: cavernous malformation in the right frontal lobe.

(***Case 1***)A 34-year-old, previously healthy woman presented with progressive visual impairment for 2 months. At presentation, her visual acuity evaluated on Snellen chart was 20/20 on the right eye and 20/200 on the left, with a bitemporal visual defect. Cranial computed tomography scans revealed a cystic mass in the suprasellar region. A homogenous hypodensity was observed, lacking intralesional calcifications (Fig. 1). The cyst, 25 mm × 15 mm in maximal dimension, showed low intensity on T1- and high intensity on T2-weighted MRI, with marginal enhancement (Fig. 2). The patient underwent endoscopic endonasal tumor resection, which resulted in gross total resection. Microscopically, the resected cyst wall revealed proliferation of squamous epithelial cells and keratin deposits, consistent with an adamantinomatous craniopharyngioma (Fig. 3). Her visual acuity examined 3 weeks after surgery improved to 20/20 on both eyes, with a marked resolution of the visual defect. Constructive interference steady-state (CISS) imaging performed before surgery and on postoperative day 24 showed a remarkable resolution of hyperintense areas in the swollen optic nerve and tract (Fig. 4).

(***Case 2***) A 36-year-old woman experienced an abrupt visual loss in the right eye. The patient had been diagnosed with cerebral CM in the right frontal lobe 11 years before, when the patient underwent MRI for transient dizziness. Periodical MRI performed 10 months prior to presentation identified an asymptomatic CM in the suprasellar region, in addition to a CM in the frontal lobe. At that time, the right optic tract appeared intact (Figs. 5A-C). The patient presented to the hospital the following day, when her visual impairment spontaneously improved from light perception. Visual acuity was 20/100 on the right eye and 20/20 on the left, with quadrantanopia in the upper temporal regions. Cerebral MRI revealed a marked enlargement of the suprasellar CM and swelling of the right optic tract. In contrast, CM in the right frontal lobe did not show any signs of recent hemorrhage (Figs. 5D-F). CISS imaging performed 3 days after the onset showed hyperintense areas in the swollen optic nerve and optic tract (Figs. 6A and B). The patient was conservatively managed with intravenous methylprednisolone (500 mg/day) for 2 days. Her visual acuity improved to 20/20 on both eyes over 3 months, with resolution of the visual defect. The CISS sequence performed 98 days after onset showed remarkable resolution of hyperintense areas in the swollen optic nerve and optic tract (Figs. 6C and D).

## Discussion

Among the 2 cases presented, the optic pathways were affected by external compression in one patient and intramedullary hemorrhage in the other. In both patients, prominent hyperintense areas were identified on initial CISS imaging, simultaneously with visual impairment. These areas showed a remarkable reduction in the post-treatment CISS sequence with improved visual function. Therefore, we assumed that the hyperintense areas found in the optic pathway may indicate congestion of the glymphatic flow coursing in the optic pathway. In addition, we believed that the reduction of these hyperintense areas may be a radiological biomarker reflecting the degree of improvement in visual impairment. These unreported, but possible speculations should be verified in further investigations. The “optic tract edema” or “optic tract edema sign” documented in previous reports as a pathognomonic finding of craniopharyngiomas might be the same as the prominent hyperintense areas observed in our patients [10,12]. Although craniopharyngiomas are grossly well-circumscribed, microscopically, the borders with adjacent neural tissues are frequently irregular with interdigitations [11]. These findings may be the basis for postoperative neurological deficits and recurrence even after total resection. Hyperintense areas in the optic pathway may appear in association with pathologies that can cause external and internal blockage of glymphatic flow in the optic pathway, leading to congestion of the flow. In our cases, recovery of visual impairment occurred earlier in the patient with craniopharyngioma than the one with CM. Direct and immediate decompression by surgery, in addition to external compression of the optic pathway, which was found in the former patient, might have contributed to the relatively short period of functional recovery. The outcome of this report was based on observations in 2 patients with different pathologies affecting the optic pathway. Further case accumulation coupled with comprehensive approaches would enable better understanding of the hyperintense areas.

In conclusion, prominent hyperintense areas in the optic pathway may indicate congestion of glymphatic flow. In addition, the reduction in such areas may be a radiological biomarker reflecting the improvement of visual impairment.

## Author contributions

All the authors contributed equally to this study.

## Ethical standards

We declare that the present study has been approved by the institution's guidelines for human research and was performed in accordance with the ethical standards laid down in the 1964 Declaration of Helsinki and its later amendments.
